# Study of Catalytic Combustion of Dioxins on Ce-V-Ti Catalysts Modified by Graphene Oxide in Simulating Iron Ore Sintering Flue Gas

**DOI:** 10.3390/ma13010125

**Published:** 2019-12-26

**Authors:** Qi Shi, Long Ding, Hong-Ming Long, Tie-Jun Chun

**Affiliations:** 1 School of Metallurgical Engineering, Anhui University of Technology, Maanshan 243002, China; 13615556646@163.com (Q.S.); Dinglongahuter@163.com (L.D.); springcsu@126.com (T.-J.C.); 2 Anhui Province Key Laboratory of Metallurgy Engineering & Resources Recycling; Anhui University of Technology, Maanshan 243002, China

**Keywords:** iron ore sintering, dioxins emission, catalytic combustion, Ce/V/Ti catalysts, graphene oxide

## Abstract

Ce-V-Ti and Ce-V-Ti/GO catalysts synthesized by the sol-gel method were used for the catalytic combustion of dioxins at a low temperature under simulating sintering flue gas in this paper. The catalytic mechanism of Ce-V-Ti catalysts modified with graphene oxides (GO) at a low temperature was revealed through X-ray diffractometer (XRD), Brunauer–Emmett–Teller (BET), transmission electron microscopy (TEM), X-ray photoelectron spectroscopy (XPS), H_2_-temperature-programmed reduction (H_2_-TPR) and Fourier transform infrared (FTIR). During the tests, chlorobenzene (CB) was used as a model reagent since the dioxins are poisonous. The results showed that introducing GO to Ce-V-Ti catalysts can improve the specific surface area and promote the CB adsorption on the surface of catalysts. Simultaneously, the Ce-V-Ti with 0.7 wt % GO support showed the high activity with the conversion of 60% at 100 °C and 80% at 150 °C. The adsorb ability of catalysts is strengthened by the electron interaction between GO and CB through π-π bond. In the case of Ce-V-Ti catalysts, Ce played a major catalytic role and V acted as a co-catalytic composition. After GO modification, the concentration of Ce^3+^ and V^4+^ were enlarged. The synergy between Ce^3+^ and V^3+^ played the critical role on the low-temperature performance of catalysts under sintering flue gas.

## 1. Introduction

Dioxin (polychlorinated dibenzofurans and polychlorinated dibenzodioxins) can cause a series of environmental problems such as photochemical smog, atmospheric ozone depletion and ground-level ozone generation, which are considered to be extremely hazardous contaminants due to serious carcinogenicity and different discharges from the body [1]. It is mainly generated from a wide range of industrial processes or incineration of municipal and medical wastes. It is said that the iron ore sintering process is one of the largest emission sources [2]. Hence, the stringent environment regulations have been imposed on the emissions from the iron ore sintering process in many countries [3,4]. Among various control methods, catalytic combustion of dioxins at low temperature (100–200 °C) attracts considerable attention for energy conservation and emission reduction during the iron ore sintering process.

Catalysts are crucial for the catalytic combustion of dioxins at low temperature. During the past few decades there are a number of catalysts developed for dioxin catalytic combustion, most of which can be classified into three types: noble metals [5,6], transition metals [7,8] and zeolites [9,10]. Although the noble metal catalysts present excellent catalytic activity at high temperature, they are susceptible to deactivation by chlorine adsorption. The activity of zeolites catalysts tends to fail gradually as the formation of polychlorinated compounds and the deposition of coke during catalytic combustion [11]. Recently, low-temperature catalytic combustion by transition metal oxides which can completely decompose these compounds has been considered as the most promising control technology owing to its low energy consumption, low cost, and good resistance to poisoning [12], especially for the effectively catalytic combustion at a low temperature from 200 °C to 400 °C without fuel combustion [13,14]. Nowadays, the catalytic combustion of dioxins has not been industrialized, so developing catalysts based on transition metal oxides with high catalytic activity at low temperature is of great significance.

It is reported that graphene oxide with oxygen functional groups is an excellent material for the functionalization of graphene and tunability of its optical properties due to its ultrathin two-dimensional (2D) sheets with 98% optical transmittance [15]. The graphene-based materials are promising for applications in supercapacitors and other energy storage devices due to highly tunable surface area, outstanding electrical conductivity, good chemical stability and excellent mechanical behavior [16].

In past research, V-Ti [17], Ce-Ti [11], V-Ce [18] and graphene-Ti [19] catalysts have been proved to have the ability of catalytic combustion of chlorobenzene (CB); However, introduced graphene oxide (GO) on the Ce-V-Ti catalysts to significantly improve the low temperature catalytic combustion of CB was never reported. In this work, a series of nanoparticles cerium vanadium titanium multi-metal oxide catalysts were prepared by the sol-gel method, and graphene oxide was introduced into Ce-V-Ti catalysts to pursue considerable catalytic conversion at low temperature under simulated sintering flue gas. In the presence of Ce-V-Ti/GO catalysts, the effect of modification on the chemical state of surface ions, oxygen vacancies and microstructure. Then, the catalytic mechanism was supposed on the results of characterizations.

## 2. Experimental and Material

### 2.1. Catalysts Preparation

Ce-V-Ti and Ce-V-Ti/GO catalysts were prepared by sol-gel method. Firstly, 3.78 g Ce(NO_3_)_3_·6H_2_O ethanol solution was added to 34.10 g Ti[O(CH_2_)_3_CH_3_]_4_ ethanol solution under stirring. Then, 0.07 g commercial industrial graphene oxide (GO, purity > 97%, Chengdu Organic Chemicals Co. Ltd., Chengdu, China) with diameter of 3–10 μm was added without further treatment. The solution of NH_4_VO_3_ (0.64 g) and oxalic acid (n_NH4VO3_:n_oxalic acid_ = 1:1) was added under stirring. The solution was slowly gelled after finishing the reaction between Ti[O(CH_2_)_3_CH_3_]_4_ and H_2_O at room temperature. The gel was dried at 110 °C for 12 h and then calcined at 450 °C for 3 h. The obtained samples were labeled as Ce-V-Ti and Ce-V-Ti/GO(x) catalysts, where x represented the weight percent of graphene oxide. TiO_2_ was the carrier, CeO_2_ was the active component, VO_X_ was the co-active component, and GO was the modified component in Ce-V-Ti/GO catalysts.

### 2.2. Catalyst Characterization

Characterization by XRD, BET, transmission electron microscope/high-resolution transmission electron microscopy (TEM/HRTEM), XPS, FTIR and H_2_-TPR was carried out. The phase structures of catalysts were analyzed by a German D8ADVANCE X-ray diffractometer (XRD) with Cu K_α_ radiation (Cu K_α_ = 0.15406 nm, Brock, Germany). The surface area and distribution of pore size were carried out using an ASAP-2020 surface analyzer from Micromeritics, Norcross, GA, USA. Transmission electron microscopy (TEM) was performed with JEM-2100. X-ray photoelectron spectroscopy (XPS) was used to analyze the oxidation states on the surface of the CeO_2_-VO_x_-TiO_2_/GO catalysts with AXIS ULTRP, which used Al Kα (1486.6 eV) radiation as the excitation source (powered at 10 mA and 15 kV). The temperature-programmed reduction (H_2_-TPR) test was carried out by an N-3000 dual-channel chromatography workstation device. FT-IR spectra were recorded at room temperature on a Fourier Transform Infrared Spectrometer (5DXC, Nicolet6700, Nicolet, Glendale, WI, USA) in the 500–4000 cm^−1^ range and a resolution of 4 cm^−1^.

### 2.3. Apparatus and Methods

**Figure 1:**
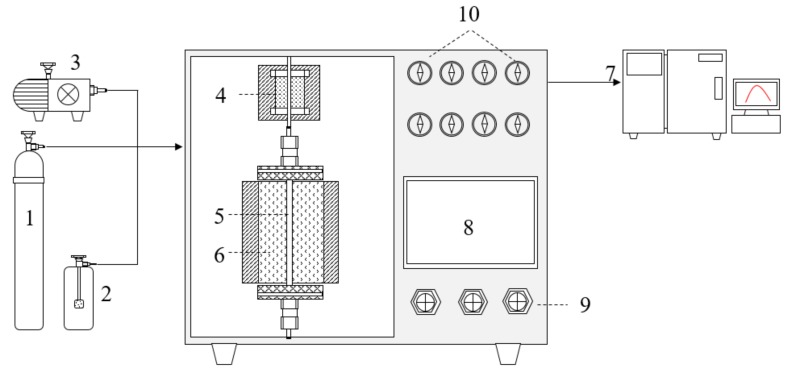
Chlorobenzene (CB) oxidation reaction device. 1 gas cylinder; 2 CB generator; 3 air pumps; 4 preheating furnaces; 5 silica tube; 6 holding furnace; 7 gas chromatograph (GC); 8 controlling panel; 9 controlling valve; 10 pressure gages.

Catalytic conversion was tested in a fixed-bed flow reactor as shown in Figure 1, in which 200 mg (grain size, 40–60 mesh) catalysts were placed in a silica tube with 3 mm inner diameter. Due to the complex structure, strong toxicity and troublesome analysis of dioxins, chlorobenzene (CB) was generally used as a model reagent in laboratories [20,21]. The total flow rate through the reactor was set at 100 standard-state cubic centimeter per minute (S ccm), corresponding to a gas hour space velocity (GHSV) 30,000 h^−1^. Feed stream to the reactor was prepared by delivering liquid CB with a syringe pump, and the injection position was cooled with ice water bath to ensure the complete evaporation of the liquid reactor feeds. The reaction flue gas was composed of 16% O_2_ and 84% N_2_, and the CB concentration was set to 100 ppm to simulate iron ore sintering flue gas. The reaction temperature was controlled with a thermocouple. The recent gases were analyzed under a given temperature by using an online gas chromatograph (GC) equipped with flame ionization detector (FID1) and SE-54 capillary column for the quantitative analysis the inlet and outlet of CB. During the CB analysis the temperature of the column and detector of GC with nitrogen as carrier gas were set up as 100 °C and 150 °C, respectively.

The conversion ratio of CB (*η*) was calculated as follows:

$$\eta =\frac{CB_{in}-CB_{out}}{CB_{in}}\;\times \;100\%$$

where the *CB_in_* and *CB_out_* were the inlet and outlet concentration of CB in the system at steady-state, respectively.

## 3. Results and Discussion

### 3.1. Catalytic Activity Analysis

**Figure 2:**
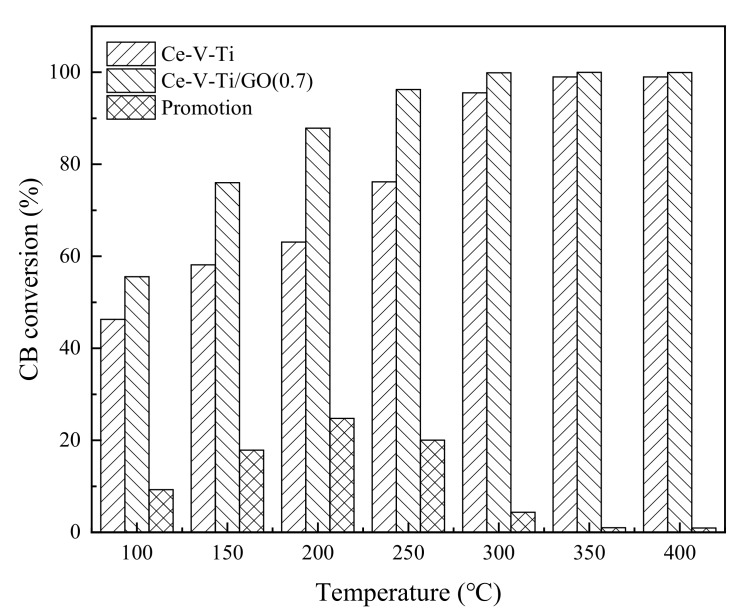
CB conversion over Ce-V-Ti and Ce-V-Ti/GO catalysts; CB: 100 ppm; gas hour space velocity (GHSV): 30,000 h^−1^; catalyst amount: 200 mg.

The catalytic activity of CB over Ce-V-Ti and Ce-V-Ti/GO catalysts is shown in Figure 2. The Ce-V-Ti catalysts showed good activity with a 90% conversion at 300 °C. After modification by GO, the activity of the catalysts increased significantly and showed high catalytic activity between 100–250 °C. The Ce-V-Ti/GO(0.7) catalysts achieved 90% conversion at 200 °C, which is 100 °C lower than Ce-V-Ti catalysts.

Research about catalytic combustion of dioxins over metal oxide catalysts is shown in Table 1. It can be seen that normal transition metal oxides catalysts showed low CB conversion at 150 °C and T_90_ are all above 225 °C. The catalytic activity at low temperature over Ce-V-Ti/GO catalysts was observably higher than those catalysts. Ce-V-Ti/GO catalysts can achieve near 60% CB conversion at 100 °C and 75% CB conversion at 150 °C, which was more suitable for catalytic combustion of dioxins from the sintering process with relatively low temperature after desulfurization (the temperature of sintering flue gas after desulfurization is about 100–150 °C).

**Figure 3:**
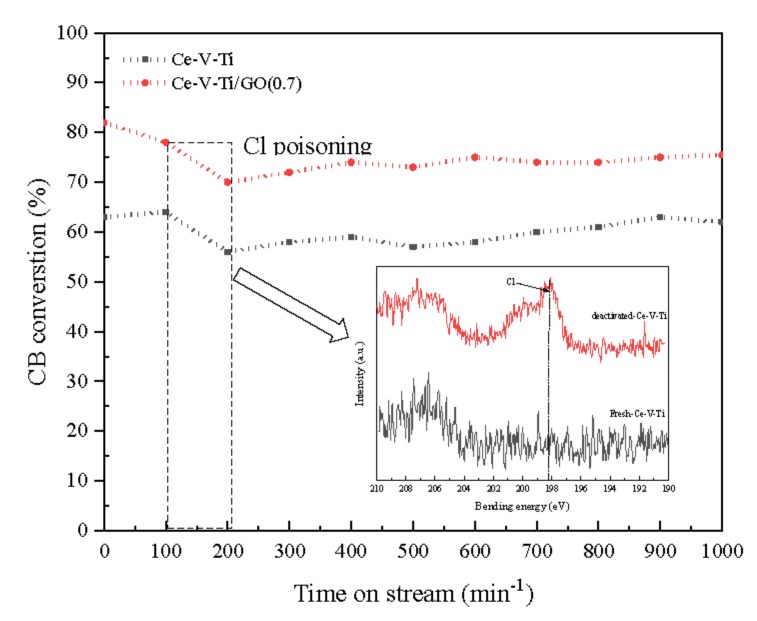
Stability for the CB catalytic combustion over Ce-V-Ti/GO at 200 °C. CB concentration: 100 ppm; GHSV: 30,000 h^−1^; catalyst amount: 200 mg.

**Figure 4:**
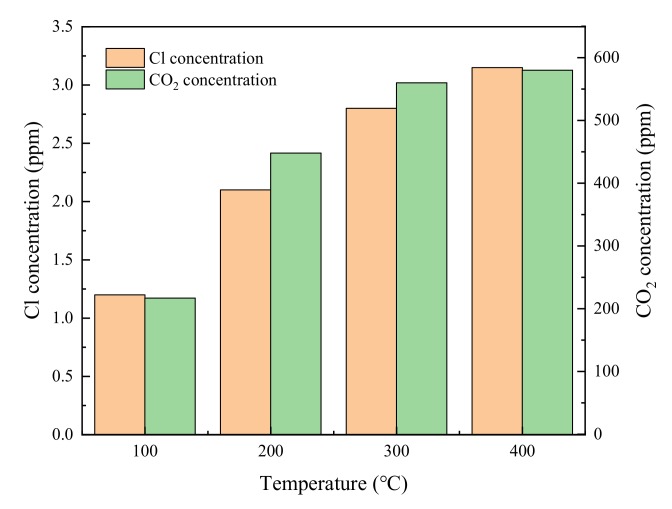
Main products of CB combustion over Ce-V/Ti-GO(0.7) catalysts. CB concentration: 100 ppm; GHSV: 30,000 h^−1^; catalyst amount: 200 mg.

For industrial applications, the stability in catalytic activity is essential for long-time operation. Figure 3 was the stability test of catalysts at 200 °C. The Ce-V-Ti/GO and Ce-V-Ti/GO(0.7) catalysts showed stable activity after 200 min and kept steady CB conversion within 1000 min. The CB conversion over Ce-V-Ti/GO and Ce-V-Ti/GO(0.7) catalysts were about 50% and 75%, respectively. Compared with the activity of fresh Ce-V-Ti/GO catalysts (Figure 2), it can be seen that the activity drops at 200 min. The deactivation is related to the strong adsorption of HCl or Cl_2_ produced during catalytic reaction [8]. In order to further confirm the deactivation of the catalyst was due to chloride poisoning, XPS was used to detect the Cl ions on the surface of the deactivated catalyst, as shown in the right corner of Figure 3. It showed that on the surface of the deactivated catalyst appeared Cl ion spectral peak located at 198.6 eV, while it cannot be detected on the surface of the fresh catalyst. The results showed that Ce-V-Ti catalysts were partially deactivated in the catalytic reaction by chlorine poisoning. The catalytic activity of Ce-V-Ti/GO and Ce-V-Ti/GO(0.7) catalysts recovered partly and kept stable as the catalytic reaction went on. CO/CO_2_ in off-gas were analyzed by online gas chromatograph (GC) equipped with a flame ionization detector (FID). Because of the sufficient oxygen in the reaction gas, no CO was detected. It indicated that CB was complete combustion during the Ce-V-Ti and Ce-V-Ti/GO catalysts combustion reaction. HCl/Cl_2_ were detected by ion chromatography (LC-2010 PLUS) after adsorption by NaOH solution. The result was analyzed strongly with HCl and Cl_2_ in Figure 4. The collected Cl species were the product of the reductive dichlorination of CB. The removal of Cl^−^ and H^+^ can combine to produce HCl, and Cl_2_ is attributed to Deacon Reaction (2HCl + 1/2O_2_ → Cl_2_ + H_2_O), which inevitably generates Cl_2_. These indicated that CB underwent a completely catalytic oxidation into CO_2_, H_2_O and HCl/Cl_2_ by the detection of CO_2_ and Cl species.

### 3.2. Textural Properties

The N_2_ adsorption-desorption isotherms revealed the textural properties of the samples. The BET surface area, pore volume, and pore size of the Ce-V-Ti and Ce-V-Ti/GO catalysts are summarized in Table 2. The specific surface area of the Ce-V/Ti catalyst was 95.7 m^2^/g and increased to 123.8 m^2^/g after adding GO. Meanwhile, the pore volume decreased from 0.29 to 0.15 cm^3^/g after adding GO, and the pore diameter remained nearly similar. The results indicated that Ce-V-Ti/GO catalysts had porous structures. Studies [30,31] have shown that a high volume of mesopores was available for the dispersion of metal oxide particles. After modification by GO, the specific area and pore volume was optimized, which was consistent with the TEM photographs. In general, large specific surface areas and porous structures are expected to improve the catalytic performance by providing more active sites, which favored the adsorption of reactants. It can account for the excellent catalytic performance of Ce-V-Ti/GO catalysts.

**Figure 5:**
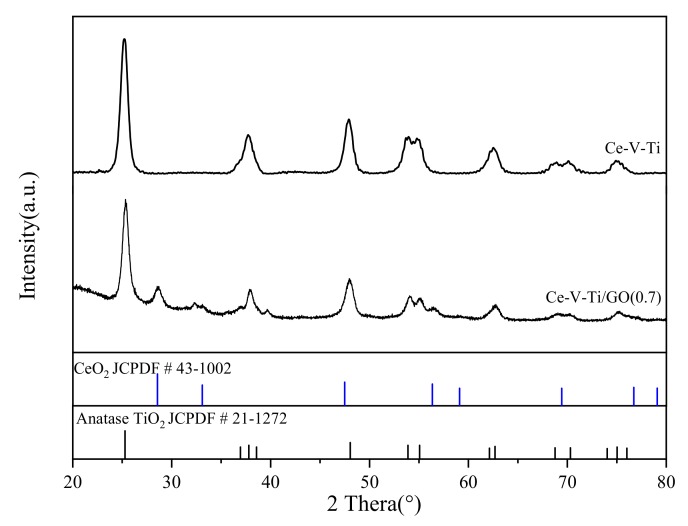
X-ray diffractometer (XRD) patterns of the Ce-V-Ti and Ce-V-Ti/GO catalysts.

XRD characterization was performed to study the internal crystal structure of the samples (Figure 5). All samples showed the characteristic peaks of anatase TiO_2_ in the in 10–80° range (25.3, 37.8, 48.1, 53.9, 55.1, 62.6, 68.7, 70.3, 74.9, and 84.6°; JCPDF # 21-1272) [32]. No obvious diffraction peaks ascribed to the crystalline cerium oxides or vanadium oxides were observed in the Ce-V-Ti catalysts, which indicated that Ce-V mixed oxides might have low crystallinity. The diffraction pattern of the Ce-V-Ti/GO catalysts contained peaks at 28.6, 33.0, 56.3, 59.0, and 76.6°, and was indexed to the face-centered cubic phase of CeO_2_ (JCPDF # 43-1002) [19]. These results indicated that the addition of GO could have promoted the crystallization of CeO_2_ by hindering the forming of a Ce-O-Ti solid solution. No diffraction peaks characteristic of carbon species was observed owing to the low carbon loading and the relatively low diffraction intensity of GO. Thus, the presence of GO was further determined by XPS.

**Figure 6:**
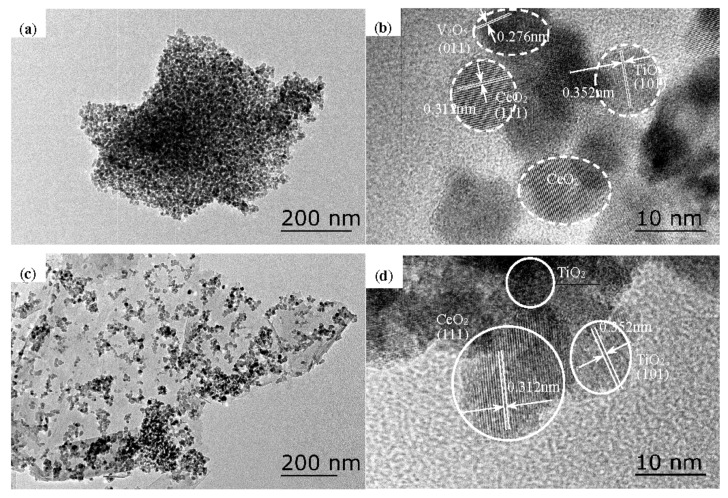
Transmission electron microscropy (TEM) images of catalysts: (**a**,**b**) Ce-V-Ti, (**c**,**d**) Ce-V-Ti/GO(0.7).

The morphology of the catalysts was characterized by TEM. As shown in Figure 6a–d, the microstructures of Ce-V-Ti and Ce-V-Ti/GO(0.7) catalysts were nanoparticles. Small CeO_2_ particles were found on the Ti-based structures. The lattice spacing (0.312 nm) matched that of the <111> crystal plane of a standard CeO_2_ sample (JCPDF #43-1002). Along with CeO_2_ particles, the TiO_2_ particles showed an exposed <101> crystal plane with a lattice spacing of 0.352 nm (anatase, JCPDF #21-1272).

Figure 6a shows TEM images of Ce-V-Ti catalysts without GO modification. These catalysts showed serious grain aggregation on the surface, which could explain the low CB conversion of catalysts. After GO modification, the Ce-V-Ti catalysts were found to contain dispersed nanoparticles on the flaky GO (Figure 6c,d). Figure 6c reveals large areas of transparent and flaky GO with many folds, and Ce-V-Ti nanoparticles were uniformly and highly dispersed on this material. The Ce-V-Ti/GO(0.7) catalysts were highly efficient in the catalytic combustion of CB at low temperature.

**Figure 7:**
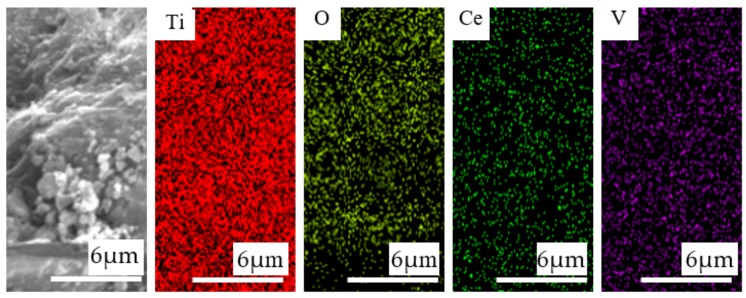
Scanning electron microscopy (SEM) images and mapping images of the Ce-V-Ti/GO(0.7) catalysts.

The TEM images also showed that the catalysts were homogeneous with no crystalline vanadium oxide phases. To further characterize the distribution of vanadium species in the catalysts, elemental mapping was conducted on Ce-V-Ti/GO(0.7) by scanning electron microscopy (SEM). As shown in Figure 7, the vanadium species were uniformly and highly dispersed on TiO_2_ in Ce-V-Ti/GO(0.7) catalysts.

### 3.3. Surface Properties

**Figure 8:**
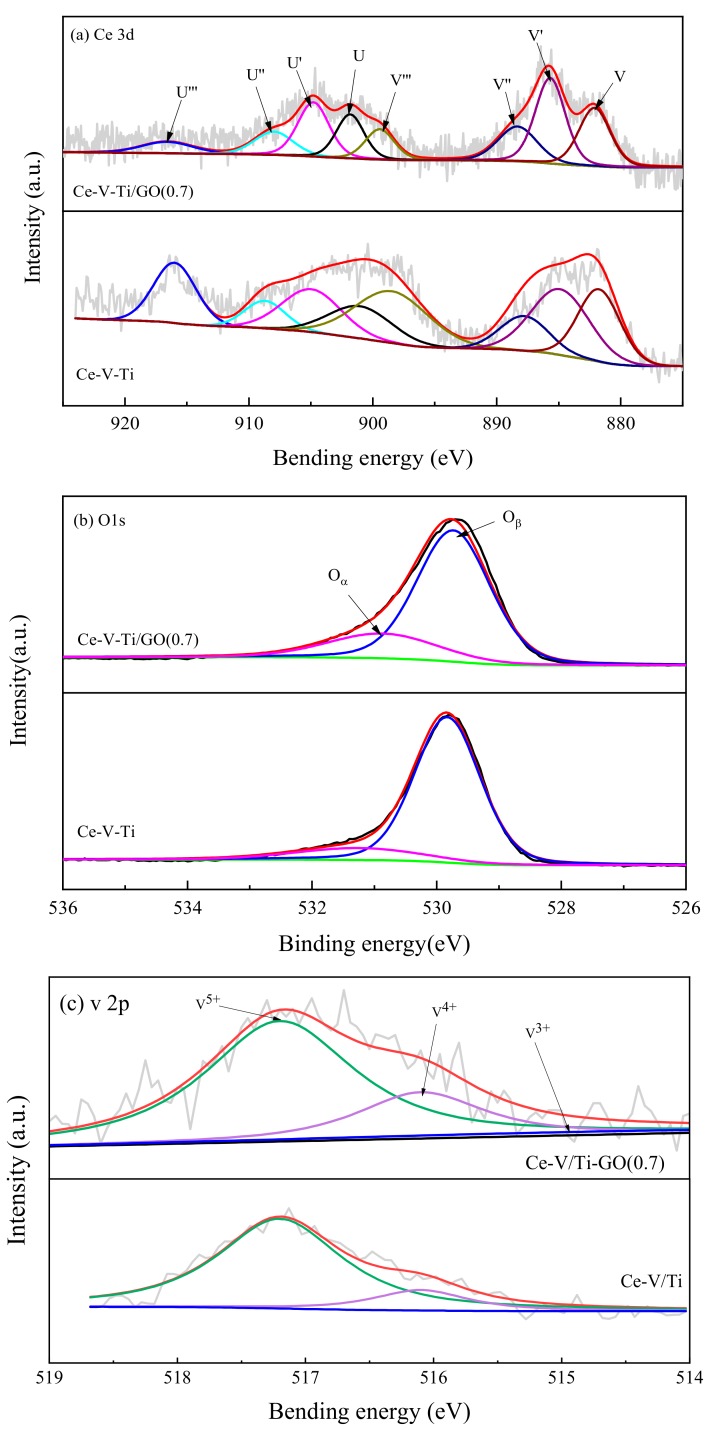
XPS spectra for: Ce 3d (**a**), O 1s (**b**), and V 2p (**c**) of the catalysts.

The atomic concentration and element chemical state on the catalyst surface was investigated by XPS. Figure 8a–c shows the Ce 3d, O 1s, and V 2p XPS spectra, respectively. The corresponding surface atomic concentrations and the relative concentration ratio of the different oxidation states are summarized in Table 3. As shown in Figure 8a, the Ce 3d orbit contained two multiples (v and u). The u, u″, u‴, v, v″ and v‴ peaks were attributed to Ce^4+^ species, while u′ and v′ were assigned to Ce^3+^ species [33,34]. There were some changes in the intensity and position of the Ce 3d bands between Ce-V-Ti and Ce-V-Ti/GO catalysts. As indicated in Table 3, Ce-V-Ti/GO(0.7) showed a significantly higher amount of surface Ce^3+^ species than the rest of the catalysts.

The fraction of Ce^3+^ ions can be stabilized by decreasing the energy of the Ce 4f levels. The reducibility of ceria was not infected by the fraction Ce^3+^ ions that were already present. As the additional electrons in Ce^3+^ occupy localized cerium 4f- states, Ce^3+^ ions did not interact with the reducibility of catalysts [4]. These Ce^3+^ species could create vacancies with one-charge imbalances and unsaturated chemical bonds on the catalyst surface, which could increase the amount of surface chemisorbed oxygen [34]. After adding 0.7% of GO, the amount of reduced Ce^3+^ ions increased. It indicated that GO changed the chemical environment and the oxidation state of Ce [35].

The O 1s spectra (Figure 8b) were deconvoluted into two contributions: one at a binding energy (BE) of 529.3–529.8 eV were assigned to lattice oxygen (O_β_) and the other BE at 531.0-531.4 eV were assigned to surface-adsorbed oxygen species (O_α_) such as O_2_^2−^ or O^−^ [36]. The peak shifts between Ce-V-Ti and Ce-V-Ti/GO revealed that the interaction between Ce-O-Ti can changed the structure and electron configurations. In the presence of GO, the BE of O_α_ shifted to lower values due to “Ce←O” electron-transfer processes through the formation of Ce-O-Ti. Moreover, the amount of O_α_ (estimated by peak area) increased with the amount of Ce species (Table 3). Thus, Ce species were mostly responsible for the onset of O_α_. The Ce-V-Ti/GO(0.7) catalysts showed the highest concentration of Ce^3+^ and a considerable amount of active surface oxygen chemically adsorbed on the vacancies (O^−^). Thus, Ce-V-Ti/GO(0.7) showed the highest activity for the catalytic combustion of CB.

Figure 8c shows XPS curves of surface vanadium species. No obvious difference was observed in the V 2p spectra. In all samples, the V 2p_3/2_ level at about 516 eV was ascribed to V^4+^ species [37]. As reported previously, oxidized vanadium was stable when deposited on titanium [32], cerium, or reduced cerium [38]. Higher amounts of V^4+^ (within a certain value) increased the amount of Lewis acid on the surface [37]. V^4+^ was oxidizable and can assist in the reduction of Ce^4+^ to accelerate the reaction catalytic cycle.

### 3.4. FTIR Spectra and H_2_-TPR Analysis

**Figure 9:**
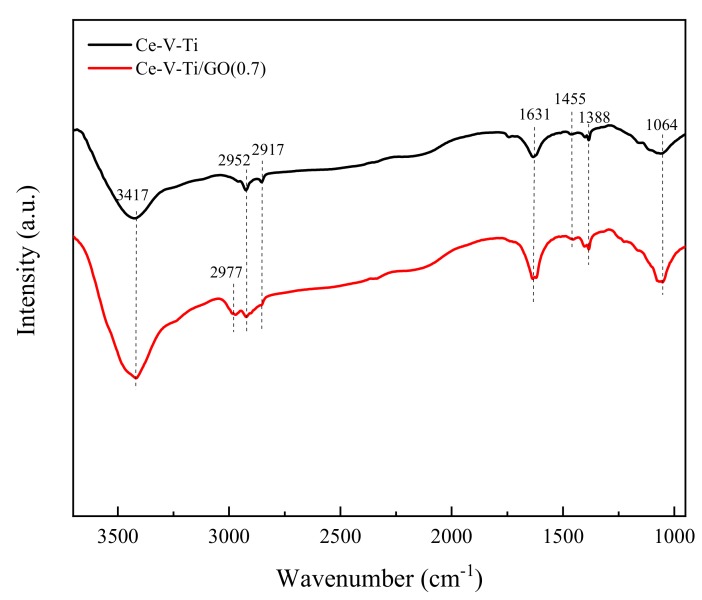
Fourier transform infrared (FTIR) spectra of the Ce-V-Ti and Ce-V-Ti/GO catalysts.

The samples were analyzed by FTIR to study the effects of GO doping on Ce-V-Ti catalysts (Figure 9). The absorption bands from 3200 to 3700 cm^−1^ were attributed to O-H stretching vibrations of hydroxyl, carboxylic, and phenolic groups [39]. The adsorption bands at 1610–1680 cm^−1^ could be attributed to C=C stretching vibrations in carboxylic or pyridine-like structures [40]. The bands at 2700–3000 and 1360–1480 cm^−1^ could be attributed to the stretching and vibration bending vibrations of aliphatic C-H groups, respectively [41]. The bands at 1050–1160 cm^−1^ were ascribed to the bending vibrations of C-O groups [18]. Figure 9 shows that surface functional groups of O-H (3417 cm^−1^), C-H (1388, 1455, 2923, and 2952 cm^−1^), C=O (1064 cm^−1^), and C=C (1631 cm^−1^) existed in Ce-V-Ti and Ce-V-Ti/GO catalysts.

The 3417 cm^−1^ peaks were more intense in the Ce-V-Ti/GO catalysts than in the GO-free catalysts. Ce-V-Ti/GO(0.7) catalysts showed a new peak at 2917 cm^−1^. The peaks at 2917 and 3417 cm^−1^ were attributed to aliphatic C-H groups and O-H stretching vibrations in hydroxyl, carboxylic, and phenolic groups, respectively. Hence, the addition of GO strengthened the intensity of aliphatic C-H groups and O-H stretching vibrations, increased the number of hydroxyls, carboxylic, and phenolic acids and favored new aliphatic C-H groups (2917 cm^−1^). As a consequence, these groups favored the adsorption of CB on Ce-V-Ti/GO catalysts, facilitating the desorption of CB [42].

**Figure 10:**
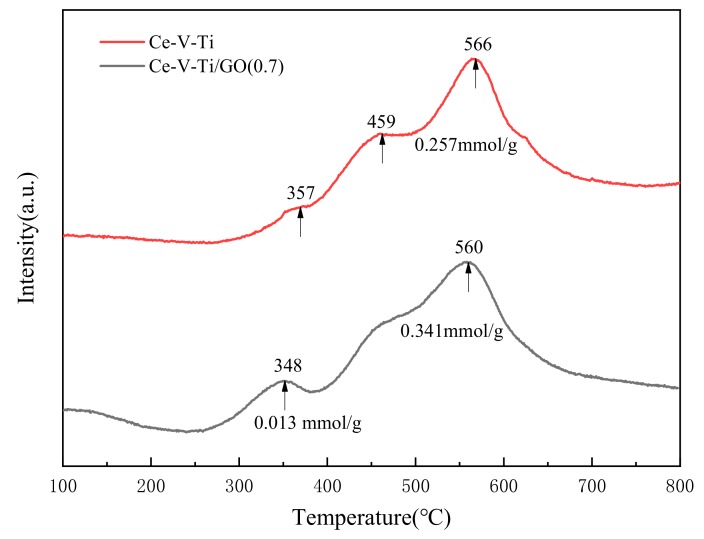
Temperature-programmed reduction (H_2_-TPR) profiles of the Ce-V-Ti and Ce-V-Ti/GO catalysts.

H_2_-TPR was used to investigate the reducibility of surface oxygen on Ce-V-Ti and Ce-V-Ti/GO catalysts within 200–700 °C (Figure 10). The H_2_-TPR plots of Ce-V-Ti catalysts mainly exhibited a two-step reduction peak of surface ceria and vanadium oxides. The reduction peak centered at ca. 460 °C might be ascribed to the reduction of VOx species [43]. The peak at higher temperatures (370–594 °C) was ascribed to the reduction of high valent ceria ions (from CeO_4_ to Ce_2_O_3_) [44]. After the addition of GO, the reduction peak of vanadium at 459 °C gradually overlapped with the reduction peak of cerium. The reduction peak of ceria slightly shifted to lower temperatures (shoulder at 565 °C–560 °C from Ce-V-Ti to Ce-V-Ti/GO catalysts). Meanwhile, the consumption of H_2_ was increased with GO addition. Further reduction reaction was facilitated with the increasing temperature, which resulted in a higher number of Ce^3+^ ions [18]. Interestingly, the reduction of Ce^4+^ to Ce^3+^ on the surface of nano-crystallites with different sizes was observed in the H_2_-TPR plots of Ce-V-Ti/GO(0.7) [45]. It indicated that the reduction of Ce^4+^ was promoted after adding GO [11]. The interaction between Ce and Ti increased the number of surface defects and active oxygen, thereby facilitating the reduction temperatures (reduction peaks of the Ce-V-Ti and Ce-V-Ti/GO were 357 and 348 °C, respectively). Therefore, the low temperature activity of CB over Ce-V-Ti catalysts with appropriate GO modification were improved by promoting the reduction of the active components.

### 3.5. Proposed Reaction Mechanism Analysis

The catalytic activity test showed that CB combustion over Ce-V-Ti catalysts was higher than most normal catalysts at low temperature. It was because the nano-TiO_2_ content of Ce-V-Ti catalysts efficiently increased the specific surface area, which could result in a high distortion extent. After being modified by GO, the low temperature catalytic activity of Ce-V-Ti catalysts was further improved.

Generally, CB combustion over the Ce-V-Ti catalyst was the Deacon reaction and followed the MVK mechanism [14]. The chlorine of CB released during catalytic combustion was adsorbed on the metal active sites (Ce^3+^/Ce^4+^ and V^3+^). Chlorine was adsorbed by nucleophilic oxygen (basic lattice oxide ions or hydroxyl groups) to form a phenolate intermediate. The catalysts adsorbed the gas-phase oxygen on the active sites to replenish the consumed oxygen. The active surface oxygen species attacked the aromatic ring, and finally oxidized CB into CO_2_ and H_2_O. To obtain deeper insight into this reaction at the molecular level, the mechanism of CB combustion over Ce-V-Ti catalysts was proposed based on the above analyses.

First, the oxidation and reduction reactions could proceed between CeO_2_ and VO_x_. Here, O* includes O_2_^−^, O^−^, and O^2−^.

$${\mathrm{CeO}}_{2}\to {\mathrm{Ce}}_{2}O_{3}+O^{*}$$

V acted as a co-catalyst in the CeO_2_/VO_x_ catalysts. During the oxidation process, oxygen could transfer from Ce-OH to VO_x_. It may promote the rate of cyclic catalytic reactions by accelerating electron transfer between Ce and V. The presence of V^3+^ improved the catalytic activity and ensured faster electron transfer during the mutual transformation of V^3+^ and Ce^4+^.

$${\mathrm{VO}}_{x}+{\mathrm{Ce}}^{4+}+O^{2-}\to V_{2}O_{5}+{\mathrm{Ce}}^{3+}$$

In the third step, according to the MVK mechanism, O* attacked CB and oxidized CB into HCl, CO_2_, and H_2_O on the active sites of the catalyst. The CB molecules were then adsorbed on the surface of the catalysts. The C-Cl molecular bond of CB break on the surface of the catalyst and degraded into an intermediate, which in turn degraded into CO_2_ and H_2_O and simultaneously produced HCl.

$$C_{6}H_{5}\mathrm{Cl}+O\to \mathrm{HCl}+{\mathrm{CO}}_{2}+H_{2}O$$

Fourth, Ce_2_O_3_ captured O from oxygen on the surface of the catalysts and was re-oxidized to CeO_2_.

Ce3++14O2→Ce4++12O2−

Hence, catalytic combustion was a process of catalytic combustion and adsorption. Improving the rate of catalytic combustion and the adsorption capacity of the catalyst could effectively improve catalytic activity. Consequently, adding GO into Ce-V-Ti catalysts can increase catalytic activity by enhancing adsorb ability and changing the adsorption mode. GO possessed a similar layered structure to graphene, and was heavily decorated with oxygen-containing groups on the planes or carbon atoms [15]. Studies have shown that the removal efficiency of dioxins, of which long axis was 1.4 nm, short axis was 0.74 nm, and the thickness was 0.35 nm [46], was related to the specific surface area and the medium pore volume of carbon materials [47]. The specific surface area was larger and the medium pore size was closer to the dioxin molecule size in Ce-V-Ti/GO catalysts after adding GO (diameter is 3–10 μm, S_BET_ is 500–1000 m^2^/g). It was consistent with the catalytic activity of Ce-V-Ti/GO catalysts.

**Figure 11:**
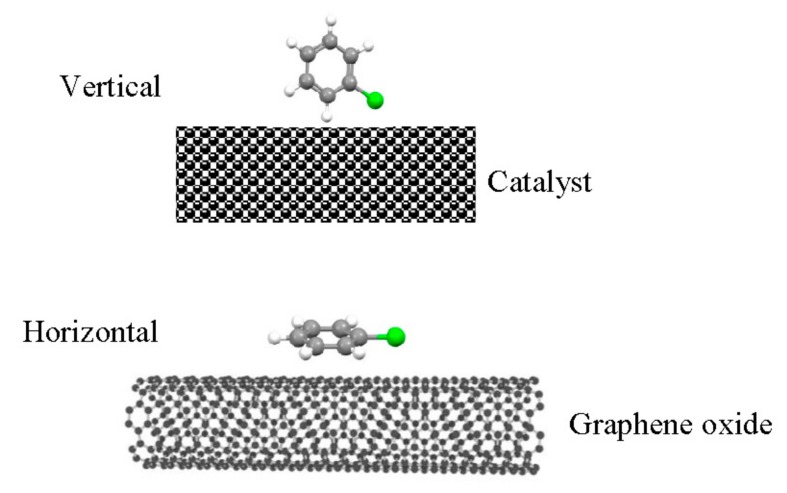
The adsorption model on the catalyst surface.

After adding GO, the adsorption mode could be changed from vertical adsorption mode to parallel adsorption mode, as shown in Figure 11. The CB molecule was vertically adsorbed to the surface of Ce-V-Ti catalysts by nucleophilic substitution (C-Cl bond breaking). It was reported that aromatic organic compounds can be adsorbed to the surface of GO through the force of π-π bond [48]. The results of FTIR show that carboxyl (-COOH) was increased with introduced GO into Ce-V-Ti catalysts. Carboxyl groups can provide electronic for π-π bonds, while CB gets electronic from π-π bonds. Consequently, the adsorption ability was enhanced by adding GO with the electron interaction. Besides, the results of XPS show that small amounts of GO can promote the amount of Ce on the surface, especially the amount of Ce^3+^ ions. The chemical interaction between Ce and Ti could improve the amount of oxygen vacancies and Ce^3+^ ions, both of which can effectively improve the adsorption of acidic sites on the catalysts surface. In addition, a certain amount of low-priced vanadium oxides appeared in Ce-V-Ti/GO catalysts, which could help promote the rate of catalytic combustion through accelerating the recycle reaction of Ce.

## 4. Conclusions

(1) The catalytic activity of Ce-V-Ti catalysts was improved significantly after modification with GO. The CB conversion over Ce-V-Ti/GO catalysts achieved 60% at 100 °C and 80% at 150 °C, which showed excellent catalytic activity at low temperature.
(2) After being modified with GO, the specific surface area and adsorb ability of CB were both enhanced. The concentration of Ce^3+^ and V^4+^ on the surface was enlarged, which corresponded with oxygen vacancies. Moreover, the adsorption mode could be changed from a vertical adsorption mode to a parallel adsorption mode by interaction of π-π bonds between CB and GO.
(3) Ce played a major catalytic role and V acted as a co-catalytic composition during catalytic combustion. The chemical interaction between Ce and Ti could improve the amount of oxygen vacancies and Ce^3+^ ions. V^3+^ could help to promote the reduction reaction.

## Figures and Tables

**Table 1:** Research about the catalytic combustion of CBs over metal oxide catalysts.

Samples	C_150 °C_	T_90_	Reaction Condition	Ref.
Ce_0.5_Ti_0.5_	0%	375 °C	1000 ppm CB; 10% O_2_/N_2_; GHSV = 30,000 h^−1^	[22]
Cr_0.75_Ce_0.25_/Ti	20%	225 °C	500 ppm CB; GHSV = 20,000 h^−1^	[23]
Mn_6_Co_2_Ce_2_	20%	325 °C	500 ppm CB; GHSV = 15,000 h^−1^	[24]
Ru (1%)/TiO_2_-CeO_2_	5%	225 °C	550 ppm CB; GHSV = 15,000 h^−1^	[25]
VO_x_ (2.1%)/CeO_2_	5%	225 °C	1000 ppm CB; GHSV = 30,000 h^−1^	[26]
Cu_0.15_Mn_0.15_Ce_0.7_O_x_	2%	255 °C	600 ppm CB; 21%O_/_N_2_; GHSV = 30,000 h^−1^	[27]
Mn (0.86)-CeLa	20%	250 °C	1000 ppm CB; 10%O_/_N_2_; GHSV = 15,000 h^−1^	[28]
MnO_X_ (0.86)-CeO_2_	20%	236 °C	1000 ppm CB; 10%O_/_N_2_; GHSV = 15,000 h^−1^	[29]

C_150 °C_: the catalytic activity at 150 °C; T_90_: the temperature of 90% CB conversion.

**Table 2:** Textural properties of the catalysts.

Sample	Surface Area (m^2^/g) ^a^	Total Pore (cm^3^/g) ^b^	Average Pore (nm) ^c^
Ce-V-Ti/GO(0.7)	123.8	0.15	6.4
Ce-V-Ti	95.7	0.29	6.5

^a^ Determined by BET surface area. ^b^ Adsorbed volume at P/P_0_ = 0.995. ^c^ Determined by desorption branch.

**Table 3:** Atomic surface compositions of Ce-V-Ti and Ce-V-Ti/GO catalysts obtained by X-ray photoelectron spectroscopy (XPS).

Sample	Atomic (%)							
	Ce	V	O	C	Ce		O	
					Ce^3+^	Ce^4+^	O_α_	O_β_
Ce-V-Ti	5.56	0.91	63.44		20	80	88	12
Ce-V-Ti/GO(0.7)	6.96	1.01	65.82	1.66	30	70	53	47
